# Biomimetic, biodegradable and osteoinductive treated dentin matrix/α-calcium sulphate hemihydrate composite material for bone tissue engineering

**DOI:** 10.1093/rb/rbad061

**Published:** 2023-06-19

**Authors:** Runying Guo, Rui Zhang, Sirui Liu, Yanyu Yang, Wenhang Dong, Meiyue Wang, Hongyan Mi, Mengzhe Liu, Jingjing Sun, Xue Zhang, Yimeng Su, Yiming Liu, Di Huang, Rui Li

**Affiliations:** Department of Stomatology, The First Affiliated Hospital of Zhengzhou University, Zhengzhou 450000, PR China; Department of Stomatology, The First Affiliated Hospital of Nanchang University, Nanchang 330000, PR China; Oral and Maxillofacial Department, Zhengzhou Stomatology Hospital, Zhengzhou 450000, PR China; Department of Stomatology, The First Affiliated Hospital of Zhengzhou University, Zhengzhou 450000, PR China; College of Materials Science and Engineering, Zhengzhou University, Zhengzhou 450000, PR China; Department of Stomatology, The First Affiliated Hospital of Zhengzhou University, Zhengzhou 450000, PR China; Department of Stomatology, The First Affiliated Hospital of Zhengzhou University, Zhengzhou 450000, PR China; Department of Stomatology, The First Affiliated Hospital of Zhengzhou University, Zhengzhou 450000, PR China; Department of Stomatology, The First Affiliated Hospital of Zhengzhou University, Zhengzhou 450000, PR China; Department of Stomatology, The First Affiliated Hospital of Zhengzhou University, Zhengzhou 450000, PR China; Department of Stomatology, The First Affiliated Hospital of Zhengzhou University, Zhengzhou 450000, PR China; Research Center for Nano-biomaterials and Regenerative Medicine, College of Biomedical Engineering, Taiyuan University of Technology, Taiyuan 030024, PR China; Department of Stomatology, The First Affiliated Hospital of Zhengzhou University, Zhengzhou 450000, PR China; Research Center for Nano-biomaterials and Regenerative Medicine, College of Biomedical Engineering, Taiyuan University of Technology, Taiyuan 030024, PR China; Department of Stomatology, The First Affiliated Hospital of Zhengzhou University, Zhengzhou 450000, PR China

**Keywords:** bone defect, treated dentin matrix, α-calcium sulphate hemihydrate, osteogenesis

## Abstract

It is still a huge challenge for bone regenerative biomaterial to balance its mechanical, biological and biodegradable properties. In the present study, a new composite material including treated dentin matrix (TDM) and α-calcium sulphate hemihydrate (α-CSH) was prepared. The optimal composition ratio between TDM and α-CSH was explored. The results indicate that both components were physically mixed and structurally stable. Its compressive strength reaches up to 5.027 ± 0.035 MPa for 50%TDM/α-CSH group, similar to human cancellous bone tissues. Biological experiments results show that TDM/α-CSH composite exhibits excellent biocompatibility and the expression of osteogenic related genes and proteins (ALP, RUNX2, OPN) is significantly increased. *In vivo* experiments suggest that the addition of TDM for each group (10%, 30%, 50%) effectively promotes cell proliferation and osteomalacia. In addition, 50% of the TDM/α-CSH combination displays optimal osteoconductivity. The novel TDM/α-CSH composite is a good candidate for certain applications in bone tissue engineering.

## Graphical Abstract

**Figure:**
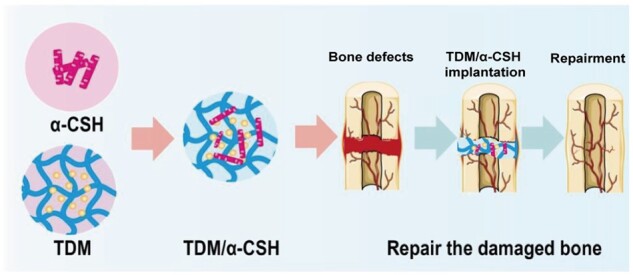

## Introduction

Bone defects caused by trauma, inflammation and tumours are commonly encountered by clinicians [1]. Bone transplantation, which encompasses autologous bone transplantation, allogeneic bone transplantation and artificial bone transplantation, is currently employed in clinical practice to treat bone defects [2]. However, autologous bone grafting has the disadvantages of causing a relatively high level of trauma, limited sources and easy absorption. Allogeneic bone transplantation has the disadvantages of a low success rate, a high infection rate and poor biocompatibility [3]. In recent years, the rapid development of materials science, tissue engineering and nanotechnology has led to the rapid development of artificial bone transplantation. It has become the development direction of future bone transplant materials [4].

The ideal bone substitute should have excellent osteoconductivity, osteoinductivity, biological activity, biodegradability and plasticity, as well as a certain amount of mechanical strength [5]. Researchers have developed various types of bone graft materials to meet clinical needs, such as hydrogel, hydroxyapatite (HA) and magnetic polymeric scaffolds [6–8]. Compared to other bone grafting materials, α-calcium sulphate hemihydrate (α-CSH) as the artificial bone transplantation has the unique combination of the following advantages: relatively simple preparation, strong plasticity, stable crystal shape, excellent biocompatibility, self-degradation, similar chemical composition to bone tissue and low cost [9]. However, the degradation of α-CSH transforms the surrounding environment to a weak acidic environment, thereby affecting the formation of new bone [2]. When α-CSH is mixed with other materials, it can play a complementary role. For example, a group of researchers combined α-CSH and HA to create a CSH–HA scaffold. The scaffold showed biocompatible, partially degradable, and exhibited a mechanical compressive strength similar to that of trabecular bone [10]. Compared to single-material bone substitutes, those made up of composite materials have more desirable effects on osteogenesis, inflammation and degradation [11–13]. However, the existing research on α-CSH composite materials remains insufficient. Thus, further investigation into the roles of the ratio and internal structure of composite materials is necessary.

Treated dentin matrix (TDM) is a biological material made by complete teeth that have been clinically extracted [14]. It has been found that TDM possesses good biocompatibility, it also contains a variety of growth factors related to odontogenesis and osteogenesis [15–18]. Instead of using common TDM preparation methods, those that have to entail the use of hydrochloric acid, freezing, calcination, and/or demineralization, we developed and implemented a gentle demineralization procedure. It only imploys ethylene diamine tetraacetic acid (EDTA) to fabricate TDM. This demineralization method dissociates the collagen fibres on the surface of the dentin matrix [19]. It causes the dentin tubules to fully open and provides a suitable release channel for related osteogenic and odontogenic active factors and proteins [19, 20]. Our previous research team also implanted human jawbone derived bone marrow mesenchymal stem cells (JBMMSC) combined with TDM subcutaneously into immunodeficient mice, we observed the formation of bone tissue like structures on the medullary side of the scaffold material, with many osteogenic lacunae distributed in these newly formed bone tissue like structures [20]. However, our previous research has shown that TDM displays a poor plasticity. It limits the applicability in the field of bone tissue engineering.

This study developed and evaluated a new type of composite material by combining granular TDM with α-CSH. After evaluating the biocompatibility and osteoinductivity of the material, it was implanted into the skull defects of Sprague–Dawley (SD) rats. It could provide a theoretical basis and new ideas for the construction and clinical application of active bone repair materials.

## Materials and methods

### Preparation of TDM/α-CSH

The experiment obtained complete teeth extracted from patients for orthodontic purposes at the First Affiliated Hospital of Zhengzhou University, with informed consent obtained from both the patient and their family members. The enamel and pulp of healthy and intact teeth were removed, exposing the dentin of the roots. The demineralized sheet-like TDM were obtained after gradient demineralization with EDTA (Solarbio, China) solution with different concentrations (17% EDTA for 10 min, 10% EDTA for 5 min and 5% EDTA for 10 min). Then, the demineralized sheet-like TDM were placed in vacuum freeze-drying machine (BILON, China) for drying, and then grounded using the high-throughput frozen tissue grinder (Servicebio, China) to obtain granular TDM. The high-throughput frozen tissue grinder needs to pre-cool to −40°C, grind the demineralized dentin slices TDM for 30 s, repeat 3–5 times every 2 min, until there are no visible particles. Treat the granular TDM with a 50-mesh cell sieve to select small size granular TDM for subsequent experiments.

Calcium sulphate dihydrate (100 g, Sigma, USA) was mixed with 10 ml of pure water and stirred continuously. The resulting mixture was then placed in a closed vessel under a pressure of 0.13 MPa and heated at a constant temperature of 123°C. Finally, the material was dried at 120°C, cooled down to 25°C, and finely ground to obtain α-CSH. The above materials were sterilized using Co-60 irradiation. It was subsequently mixed with α-CSH at different TDM mass ratios of 0%, 10%, 30%, 50% and 70%. Then, the TDM/α-CSH powder was mixed with deionized water at a liquid-to-solid ratio of 0.4:1 (ml/g) to obtain the α-CSH, 10%TDM/α-CSH, 30%TDM/α-CSH, 50%TDM/α-CSH and 70%TDM/α-CSH materials. Subsequently, cylinders test specimen (5 mm × 5 mm × 1 cm) were fabricated for material characterization testing, whereas disks test specimen (5 mm × 5 mm × 5 mm) were made for *in vivo* experiments. Then, the composite materials were immersed in α-minimal essential medium (α-MEM, Hyclone, USA) at a solid-to-liquid ratio of 0.2:1 (g/ml) and then placed in a 37°C, 5% CO_2_ incubator for 3 days. The leaching solution of each group of materials was obtained for subsequent cell experiments.

### Compressive strength of materials

Each material specimen was placed in an electronic universal testing machine (UTM6104, China), which was operated at a compression speed of 0.5 mm/min. Each group was analysed in triplicate.

### Scanning electron microscopy observation

Fractured treatment was performed on the sheet-like TDM and composite samples (10%TDM/α-CSH, 30%TDM/α-CSH and 50%TDM/α-CSH) in liquid nitrogen to obtain smoother cross-sections. The composite material specimen, sheet-like TDM with cross-sections, and granular TDM were placed in an oven to dry prior to being sprayed with gold in a vacuum coater (Leica EM ACE600, Germany). Each material specimen was then scanned using field-emission scanning electron microscopy (SEM, Zeiss Auriga, Netherlands).

### X-ray diffraction analysis

The prepared material specimens were ground into a powder; then, the powder was placed in an X-ray diffraction (XRD) system (Empyrean, Netherlands) to enable analysis of their crystalline phase composition. The scanning parameters were as follows: a 2θ range of 10–70°, and scanning speed of 0.328°/s, scanning voltage of 45 kV, and scanning current of 40 mA.

### Fourier-transform infrared spectrometry analysis

The materials were ground into a powder. The tableting method was applied to compress the material and KBr powder. The resulting film specimens were subjected to test using a Fourier-transform infrared (FTIR) spectrometer (STA8000-Frontier, Netherlands).

### X-ray-photoelectron-spectroscopy analysis

X-ray-photoelectron-spectroscopy (XPS) technology were used to analyse the chemical properties of the surface of substances such as the composition, elements and valence states. In this study, the elemental composition of the surface of each group of materials was analysed by XPS (Thermo Fisher Scientific, K-Alpha+).

### Haemolysis test

Each material mixture sample were incubated with diluted rabbit blood in a 37°C water and bath for 60 min. Following centrifugation at 1000 rpm for 5 min, 50 µl of supernatant was collected, and the optical density (OD) values for the supernatants of each group were measured at 545 nm. Haemolysis rate was calculated from the following:


(1) $$\text{Haemolysis rate}\;\left(\%\right)=\left({\text{OD}}_{0}-{\text{OD}}_{1}\right)/{\text{OD}}_{2}-{\text{OD}}_{1})\times 100\%,$$

OD_0_: Sample optical density, OD_1_: Negative control optical density, OD_2_: Positive control optical density.

### Degradation experiment

The mass of the specimen was calculated and recorded as M_1_. Each specimen was then immersed and allowed to soak in 10 ml of simulated body fluid (SBF, Shanghai Yuanye Bio-Technology, China) and replace the SBF every 3 days. Samples from each group were placed in an oven. The weight of the dried material treated in oven is recorded as M_2_. The degradation ratio was calculated using the following:


(2) $$\text{Degradation}\;\left(\%\right)=\left(M_{1}-M_{2}\right)/M_{1}\times 100\%.$$

### Water contact angle test

The contact angle was tested by a drop shape analyser (DSA25, Kruss, Germany) to determine the hydrophilicity of the material. This process was repeated three times for each sample.

### Isolation and culture of BMSCs

The femur and tibia of 6-week-old SD rats were soaked in penicillin–streptomycin (Solarbio, China) for 30 min. Next, we cut both ends of the bone and applied α-MEM to rinse the bone cavity to extract all of the bone marrow. The bone marrow was resuspended in complete medium and then placed in the 5% CO_2_ incubator at 37°C. The P3 generation cells were used for subsequent experiments.

### BMSCs identification

BMSCs were cultured with adipogenic induction liquid (Solarbio, China) and osteogenic medium (10 mM β-sodium glycerol phosphate, Sigma, USA; 100 nM dexamethasone, Sigma; 50 mg/ml ascorbic acid, Sigma). After 14 days of adipogenic induction, the lipid droplets were observed under a microscope (Nikon, Japan). After 28 days of osteogenic induction, the appearance of the mineralized nodules was observed.

CD29 (1:100), CD31 (1:100), CD73 (1:100) and CD90 (1:100) flow cytometry antibodies all from BD Biosciences (USA) were added to the BMSCs and incubated on ice for 30 min in the dark. After applying flow cytometry (BD Biosciences) to the cells, FlowJo v10 software was used to analyse the data.

BMSCs were fixed with 4% paraformaldehyde. Cells were permeabilized with 0.1% Triton-100 (Solarbio, China) and then blocked with 10% goat serum. Primary antibodies (anti-vimentin, 1:1000, Abcam; anti-CK-14, 1:1000, Millipore, USA) were added to each well, and the cell culture plate was placed in a 4°C refrigerator overnight. The next day, the fluorescent secondary antibody was added. 4ʹ,6-Diamidino-2ʹ-phenylindole (DAPI, Solarbio, China) was added to counter-stained the cell nuclei. The cells were placed in an inverted fluorescence microscope (Nikon, Japan) for observation.

### Cell proliferation assay

BMSCs were seeded in a 96-well flat-bottom plate. After the cells were cultured for 1, 3, 5 and 7 days, 10 µl of the Cell Counting Kit-8 (CCK-8, Dojindo, Japan) reagent was added to each well. The OD value for each well was measured by using 450-nm spectrophotometry (Molecular Devices, USA).

### Osteogenic differentiation evaluation *in vitro*

After being cultured with for the extracts of each material supplemented 7 days. Then, real-time polymerase chain reaction (qRT-PCR) was performed to detect the expression levels of RUNX2, ALP and OPN genes.

After being cultured with for the extracts of each material supplemented 14 days, the proteins of BMSCs were extracted. The extracted proteins were transferred to a polyvinylidene difluoride (PVDF) membrane (Millipore, USA). Then, the PVDF membrane was blocked with 5% BSA; the following primary antibodies were added and allowed to settle overnight: GAPDH (1:10 000, Zenbio, China), ALP (1:1000, Santa, USA), RUNX2 (1:1000, Abways, China) and OPN (1:1000, Santa, USA). Goat anti-rabbit and goat anti-mouse secondary antibodies (1:10 000; Abways, China) were also added. The bands were detected with ECL Plus kit (Beyotime, China).

### Osteogenic differentiation evaluation *in vivo*

All animal experiments were performed in accordance with Animal Research: Reporting of *in vivo* Experiments (ARRIVE) guidelines, and all animal experiment protocols were approved by the Biomedical Ethics Committee of Zhengzhou University (2022-KY-0016-002). Seven-week-old male SD rats were purchased from Jinan Pengyue Laboratory Animal Breeding Co. Ltd. The pre-prepared sheet material (with the size of 5 mm × 5 mm × 1 mm) was positioned at the surgical area. The rats were euthanized at 6 weeks after the operation. The samples of the skull and major organs (heart, kidneys, liver, lungs and spleen) were extracted and fixed in paraformaldehyde. The organ specimens were stained with haematoxylin–eosin (H&E). The skull specimens were subjected to H&E, Masson’s tri-chrome and histochemical staining. Observations of the rat skull specimens were completed using a micro-computed tomography system (micro-CT, Bruker-microCT, Belgium).

### Statistical analysis

Graphpad Prism 8.0 was used to perform data statistics and analysis. Three sets of data were collected for each sample; the mean ± standard deviation was applied for statistical analysis. The following statistical analysis tests were performed: the corrected t-test, t-test and one-way analysis. The statistical significance was set at *P* < 0.05.

## Results

### Characteristics of TDM/α-CSH composite

**Figure 1.**
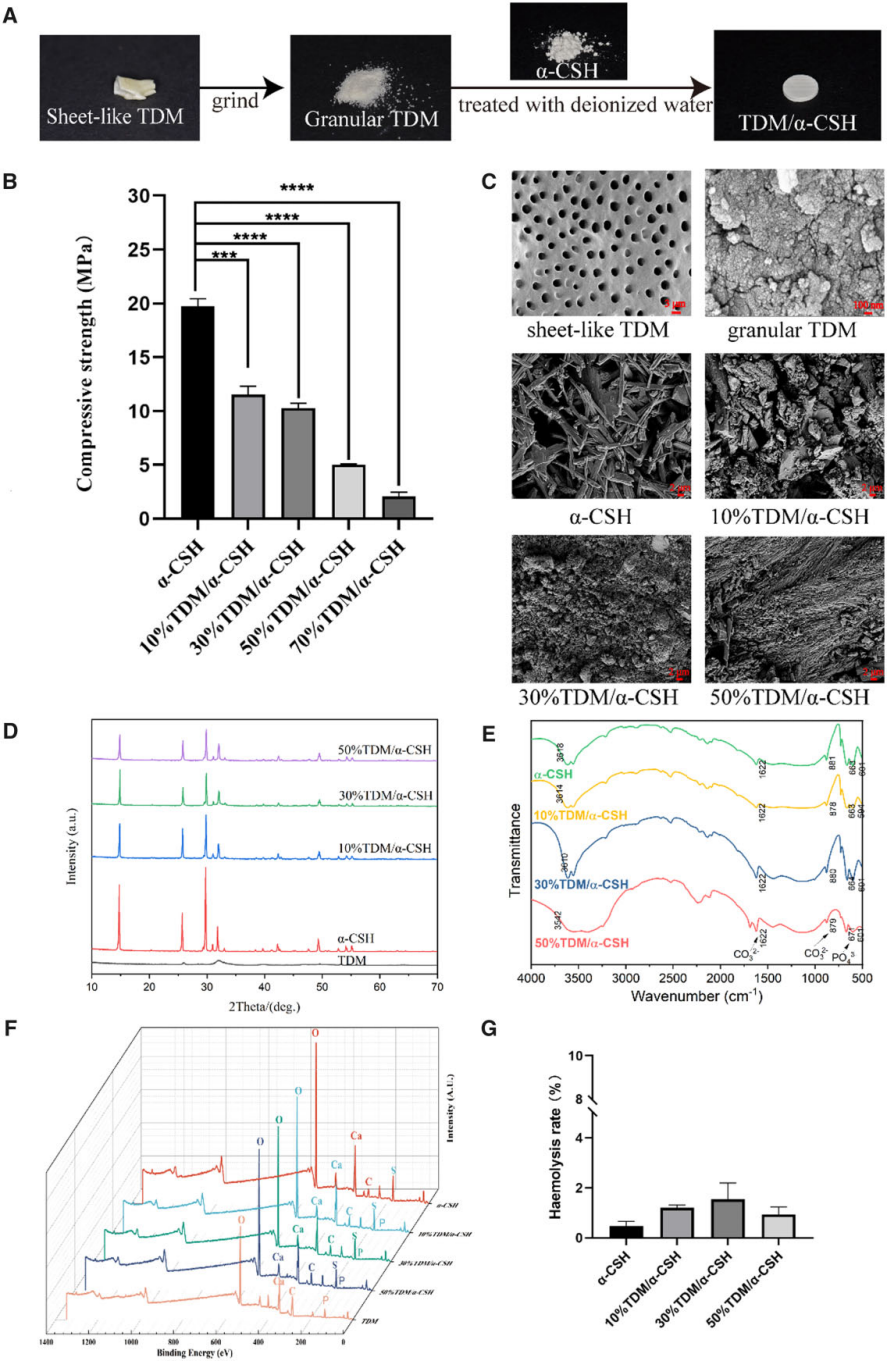
Characteristics of TDM/α-CSH composite. (**A**) Schematic illustration showing the fabrication of TDM/α-CHS. (**B**) Compressive stress of the materials. (**C**) SEM microstructure results. (**D**) XRD patterns. (**E**) FTIR spectra. (**F**) XPS result. (**G**) Haemolysis test of different materials; the results are expressed as the mean ± standard deviation of three independent experiments. ***P < 0.001, ****P < 0.0001 relative to the α-CSH group.

Benefiting from the advantages of the TDM and α-CSH, TDM/α-CSH composite exhibits unique material properties, such as biocompatibility and biodegradablility. The manufacturing illustration is shown in Fig. 1A. Bone tissue always sustains a certain amount of mechanical force in the body. Therefore, there are certain requirements for the mechanical strength of bone grafting materials [2]. The results demonstrate that as the granular TDM content in the composite material increases, the compressive stress of the TDM/α-CSH composite material significantly decreases (Fig. 1B). The compressive stress of 70%TDM/α-CSH group is only 2.072 ± 0.227 MPa, and the plasticity of 70%TDM/CSH disappears after contact with simulated body fluid (Supplementary Fig. S1). In clinical applications, it is difficult to meet the requirements of mechanical strength and plasticity [5, 21]. The compressive stresses of the other groups added TDM were 11.550 ± 0.456 (10%TDM/α-CSH), 10.270 ± 0.278 (30%TDM/α-CSH), 5.027 ± 0.035 (50%TDM/α-CSH), respectively, which significantly matched the cancellous bone mass (2–12 MPa) and were used for subsequent experimental studies [22].

The structure of the bone graft material can have a certain impact on the formation of new bone [23]. To study the microstructure of composite materials, SEM was used to observe the crystal morphology and particle size of the material surface (Fig. 1C). The results show that the α-CSH material had a short and loose porous rod structure [24]. Following EDTA gradient demineralization, the collagen fibres in part of the matrix of the sheet-like TDM could be found to be exposed. It may have contributed to the release of growth factors related to osteogenesis [20]. As the TDM particle content increases, the volume of short rod-like structures gradually decreases. Conversely, the volume of the round particles and 3D network structure increases. Additionally, the cross-section of composite material becomes more porous. This 3D porous structure is believed to contribute to the formation of new bone [25].

XRD was used to analyse the crystalline phase composition of materials [26]. The results in Fig. 1D show that no new diffraction peaks appear, indicating that the TDM/α-CSH composite material does not produce new substances. FTIR spectrometry was used to analyse the surface functional groups and spatial conformations of materials [27]. The FTIR detection results are consistent with those of XRD analysis, further verifying the main component of the TDM/α-CSH. The increasing the TDM content does not lead to any significant shift in the characteristic peaks of the composite material (Fig. 1E). Supplementary Figures S2 and S3 show that the main component of the TDM was HA. The XPS (Fig. 1F) results show that the main element of α-CSH is Ca, O and S [28] and the main elements of the TDM are Ca, C, O and P [29]. The TDM/α-CSH formed by the combination of the two materials is mainly composed of Ca, C, O, S and P elements. Through XRD, FTIR and XPS analysis, it can be concluded that no new substances were produced during the formation of TDM/α-CSH composite materials, suggesting that each sample group is purely a physical mixture of α-CSH and TDM.

The haemolysis test was used to evaluate the blood compatibility of each sample. In accordance with previous reports, in this study, a value below 5% means that the material is acceptable [30]. As shown in Fig. 1G, the haemolysis rate for each material specimen is less than 5%. The reason may be that both α-CSH and TDM have good biocompatibility [14, 31, 32]. Additionally, the results of the degradation experiment (Supplementary Fig. S4) demonstrate that the degradation rate of the composite material increases as the TDM content increases. The degradation of the composite materials could provide more space for new bone growth, as well as facilitate the implantation of new bone tissue [5]. The contact angle was used to detect the hydrophilicity of the samples. A hydrophilic material surface can effectively promote cell infiltration and adhesion [33]. The contact angles of each group of materials indicate that all samples present hydrophilic property, indicating that all samples perform hydrophilic properties (Supplementary Fig. S5).

### Identification of BMSCs

**Figure 2.**
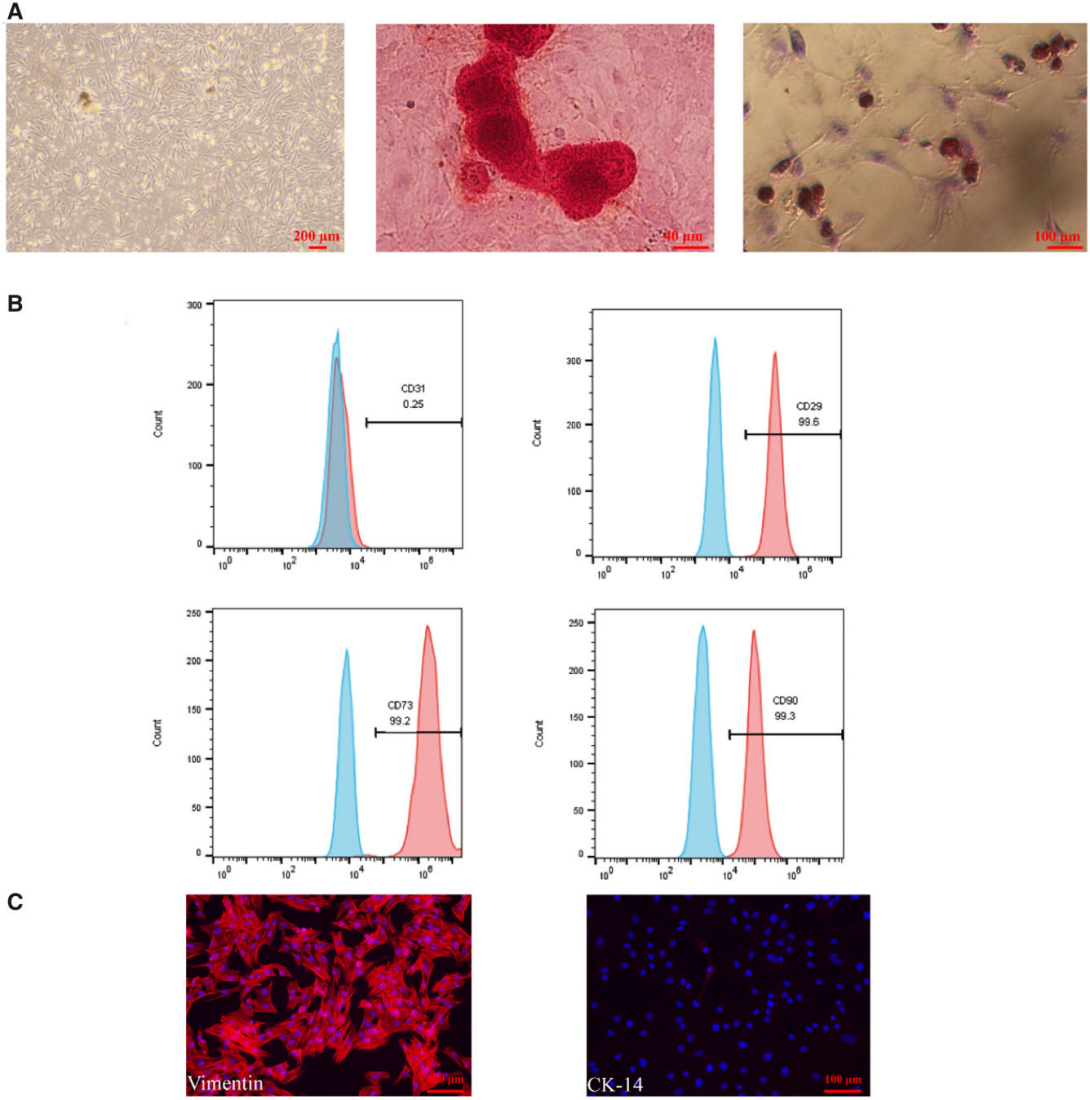
Identification of BMSCs. (**A**) BMSCs are scattered, uniform in shape and fibrous. Mineralized nodules and lipid droplets were found when the BMSCs were cultured in osteogenic or adipogenic medium, respectively. (**B**) Flow cytometry results showed that the BMSCs were positive for CD 29, CD73 and CD 90 and negative for CD31. (**C**) Immunofluorescence results showed that BMSCs were positive for vimentin and negative for CK-14.

As shown in Fig. 2A, the primary BMSCs present a long and thin polygonal shape. After subjecting the BMSCs to osteogenic induction for 21 days, mineralized nodules could be clearly observed in the Alizarin Red stained results. Additionally, 14 days after the induction of lipogenic staining, the results of Oil Red O staining reveal the appearance of lipid droplets. Alternatively, the flow cytometry results show that the mesenchymal surface markers CD29, CD90 and CD73 are positive, whereas the endothelial cell surface marker CD31 is negative (Fig. 2B). The immunofluorescence results (Fig. 2C) confirm the presence of vimentin (a mesenchymal surface marker) and the absence of the epithelial cell marker CK-14. These results indicate that the cells we obtained are of mesenchymal origin, and that they have the ability to differentiate in many ways.

### TDM/α-CSH complex effects on BMSC proliferation and differentiation

**Figure 3.**
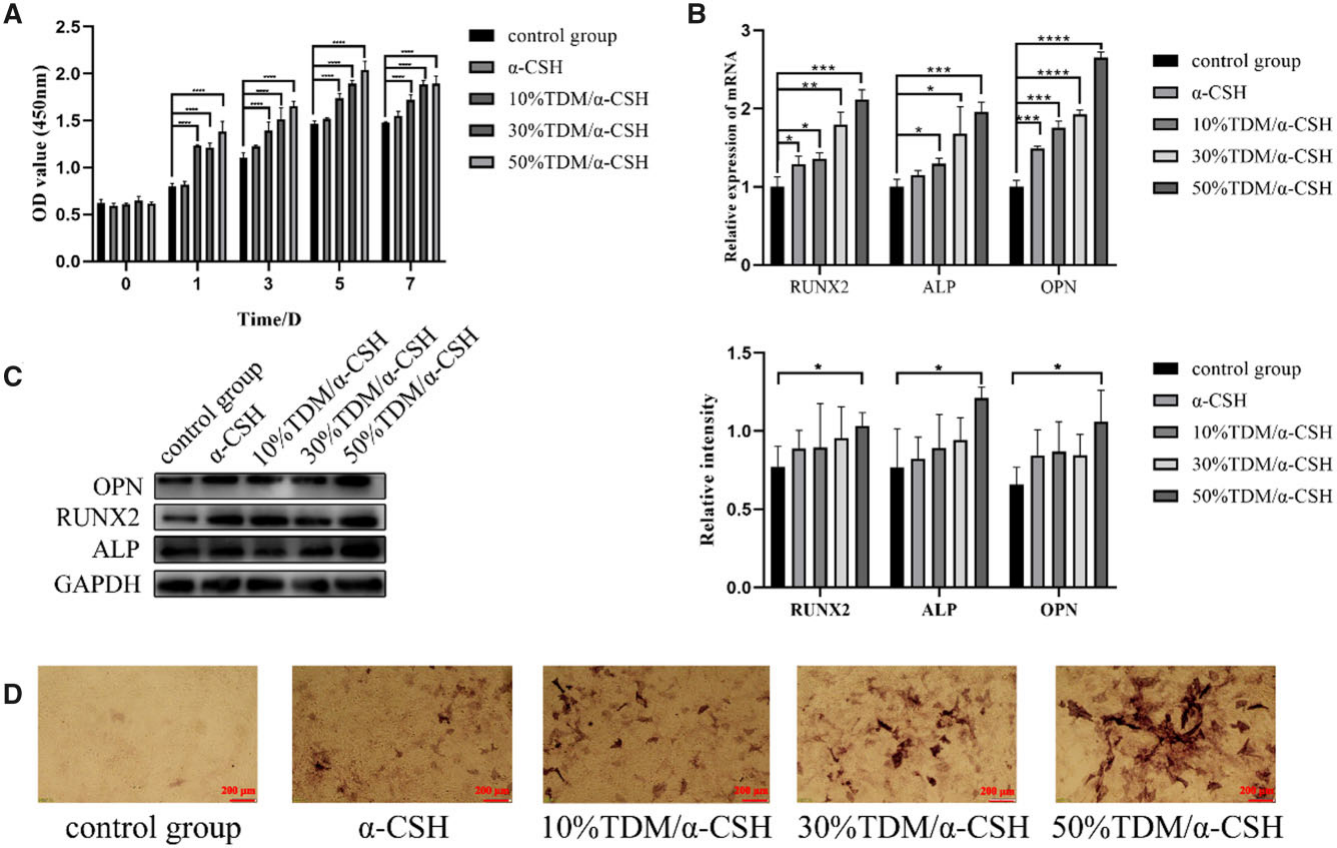
Effects of TDM/α-CSH composite extracts on proliferation and differentiation of BMSCs *in vitro*. (**A**) Illustration of the effects of the material extracts on the growth and proliferation of the BMSCs; the expression of osteogenesis-related genes and proteins treated with the designated material extract was assessed by (**B**) qRT-PCR and (**C**) western blot analysis. The results are expressed as the mean ± standard deviation of three independent experiments. (**D**) ALP staining detected ALP activity. *P < 0.05, **P < 0.01, ***P < 0.001 and ****P < 0.0001 relative to the control group.

As shown in Fig. 3A, BMSCs show obvious proliferation as TDM content increasing at different culture time. It may be contributed to the growth factors rich in TDM that can promote cell proliferation [34]. The results shown in Figs 1G and 3A indicate that TDM/α-CSH composite presents excellent biocompatibility, and thus provides theoretical support for the implantation of the composite material in SD rats. The qRT-PCR and WB results reveal that the expression of each of the osteogenesis-related indicators ALP, OPN and RUNX2 grows as TDM content increases (Fig. 3B and C). In contrast, 50%TDM/α-CSH group exhibits a significant increase of bone formation-related genes and proteins, indicating a likely positive effect of 50%TDM/α-CSH group on osteogenesis. The results of the ALP staining also support this conclusion (Fig. 3D).

### Effects of TDM/α-CSH complex on bone defect regeneration in SD rats

**Figure 4.**
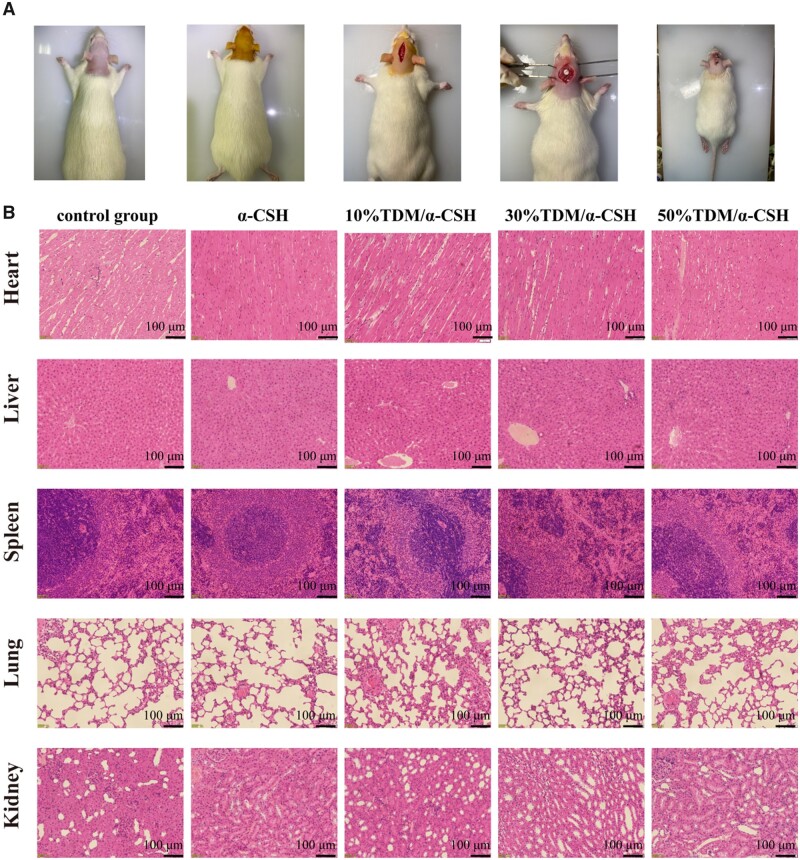
Preparation of SD rat skull defect model (**A**) and H&E staining of major organs (**B**).

TDM/α-CSH materials were used to repair calvarial defects in SD rats to further verify the osteogenic inductive ability and *in vivo* biocompatibility (Fig. 4A). A histological examination of the liver, heart, spleen and kidney biopsies does not reveal any observable differences between the TDM/α-CSH and the control groups (Fig. 4B), indicating no cytotoxicity in the rats after treatment with the composite materials compared with the healthy rats. Micro-CT was used to quantify the healing of the rat skull defects to observe the osteogenic properties of the composite material [35].

**Figure 5.**
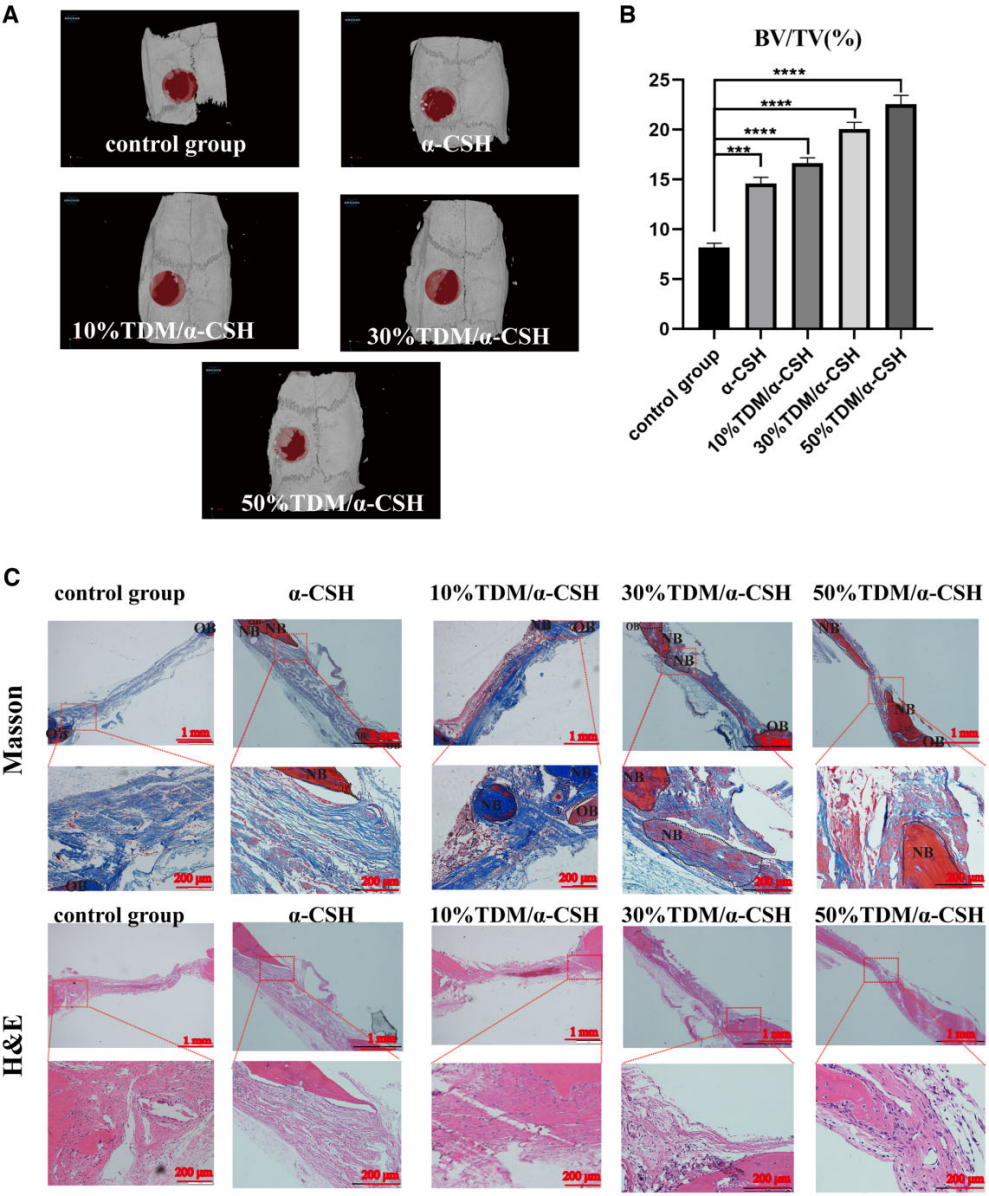
Micro-CT results after 6 weeks and Masson and H&E staining of samples. (**A**) 3D micro-CT reconstruction images of 6-week post-defect creation in SD rat skulls; (**B**) BV/TV statistical analysis results for each group. (**C**) H&E and Masson staining. The results are expressed as the mean ± standard deviation of three independent experiments. OB, primordial bone; NB, newborn bone. ***P < 0.001 and ****P < 0.0001 relative to the control group.

It can be seen in Fig. 5A that the control group performs the least amount of new bone formation. Conversely, there is significantly more new bone formation for the 50%TDM/α-CSH group. A bone volume fraction (BV/TV) quantitative analysis of each specimen shows that the 50%TDM/α-CSH sample exhibits the strongest positive effects on bone formation (Fig. 5B).

To further evaluate the osteogenic properties of the composite materials, histological and immunohistochemical staining were performed. The results of H&E and Masson staining in Fig. 5C reveal that the control group presents minimal blood vessel formation and bone tissue (i.e. only a thin layer of fibrous tissue).

**Figure 6.**
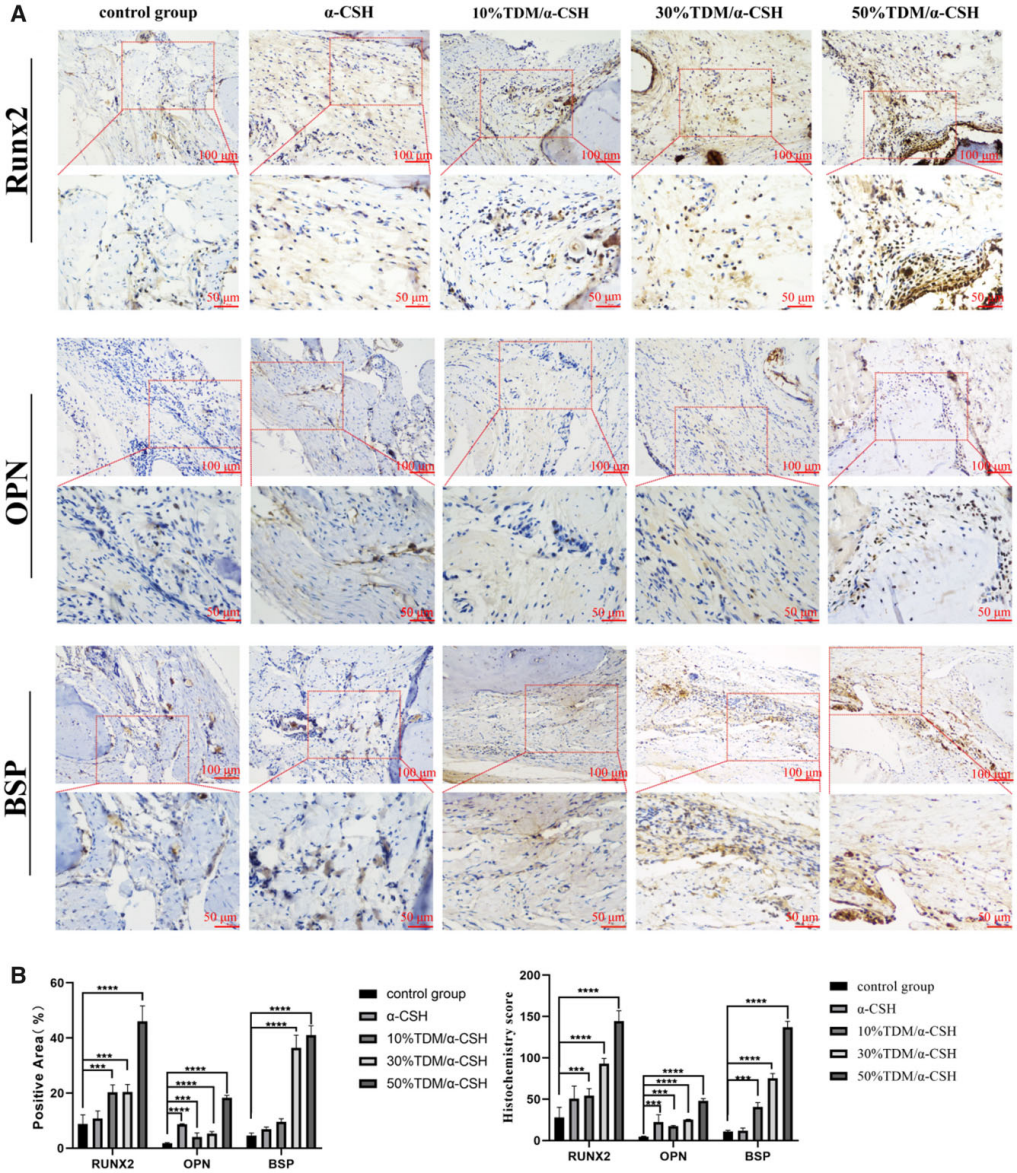
Immunohistochemical results for skull defect specimens. (**A**) RUNX2, OPN and BSP immunohistochemical results. (**B**) Histochemistry score and positive area results for RUNX2, OPN and BSP tests. ***P < 0.001 and ****P < 0.0001 relative to the control group.

Furthermore, examination of the defect site of each experimental group confirms that bone tissues grow to varying degree. Moreover, no inflammatory scar tissue, or localized acute or chronic inflammatory cell infiltration is observed (Fig. 6A). Among all of the groups, the 50%TDM/α-CSH group shows more mineralized bone tissues. The immunohistochemical staining results that tested for RUNX2, BSP and OPN supported this finding (Fig. 6B).

## Discussion

In this study, a new type of TDM/α-CSH composite material was prepared to figure out the problem of clinical bone defect repair. TDM/α-CSH composite material performs self-solidification properties after encountering water. Further, TDM is rich in a variety of osteogenic growth factors, and displays good biocompatibility, degradability and osteoinductivity. The inorganic components of human bone tissue are mainly calcium and phosphorus. TDM/α-CSH composite containing these bioactive elements. It could provide cells with surrounding environment similar to that of human bone tissues.

Generally, α-CSH slowly releases Ca^2+^ ions after degradation. It can provide a source of calcium during the formation of new bone and promote cell proliferation [36, 37]. The primary component of the TDM material is HA, which has a very similar inorganic composition to that of bones. Bone graft materials based on hydroxyapatite have been widely used in basic experimental research and clinical procedures [38, 39]. Through XRD and FTIR, we have concluded that in the newly prepared TDM/α-CSH materials, no new substances are produced, but a physical mixture of α-CSH and TDM. It can be inferred from the tissue structure that the composite has good biocompatibility and can promote healthy bone repair. Our subsequent experiments verified this conjecture.

After studying the biological properties of various materials, we found that 50% TDM/α-CSH exhibits the best osteoinductivity. The reason may be related to TDM. In our previous experiments, TDM materials release biologically active factors into the surrounding environment at a certain rate [19]. It is well-established that some growth factors, bioactive proteins, and inorganic elements with osteogenic ability have been combined with artificial bone grafting materials to develop materials with the desirable characteristics of different components and to enhance bone induction ability [40–42]. The rate of degradation of the composite material increases with TDM increasing. Thereby, more granular TDM rich in factors is exposed to the bone defect. In addition, the degradation of the composite materials can provide space for new bone to grow, as well as facilitate the implantation of new bone tissues [43]. The reason for the highest osteoinductivity exhibited by 50% TDM/α-CSH may also be related to the network structure what we observed in SEM results. The network structure serves to facilitate the transportation and output of nutrient metabolism waste. It also provides an excellent matrix that promotes cell adhesion, growth, new bone formation and vascularization in the defect area [44, 45].

However, although the increase of TDM can enhance the osteoinductive properties of the composite material, it reduces the compressive strength of TDM/α-CSH composite. The main component of our TDM is HA crystals. However, the compressive strength of the HA/α-CSH biphasic ceramic bone graft prepared by Chang *et al*. [46] was higher than that of the bone graft comprising only α-CSH. We speculate that this inconsistency may be attributable to the following facts: (i) TDM does not solidify when exposed to water and (ii) the mechanical strength of the TDM/α-CSH material is related to the amount of calcium sulphate dihydrate (CaSO_4_·2H_2_O) that is produced by the hydration reaction between α-CSH and water [47]. CaSO_4_·2H_2_O crystals are known to be cross-linked into a network, forming a dense solid with high compressive strength [48]. However, addition of the TDM component materials induces the formation of more and larger pores within the post-cured composite material, resulting in a decrease in the mechanical strength of the composite material. Bone tissue always sustains a certain amount of mechanical force in the body, and there are certain requirements for the mechanical strength of bone grafting materials [2]. Therefore, our next goal is to adjust the mechanical strength of composite materials under the premise of ensuring biological activity, and design materials with more biological functions.

## Conclusions

A novel TDM/α-CSH composite material with improved biomimetic, biodegradable and osteoinductive has been developed by using TDM in combination with α-CSH. The TDM/α-CSH composite material, especially 50% TDM/α-CSH material, significantly promotes BMSCs differentiation, mineralization, and proliferation *in vitro*. Consistently, *in vivo* data also indicates the TDM/α-CSH scaffolds could strongly support bone/ossicle formation. These results prove that the 50%TDM/α-CSH composite material has strong potential as a scaffold to promote bone defect repair.

## Supplementary Material

rbad061_Supplementary_Data
