# Supplementary material for: Biomimetic, biodegradable and osteoinductive treated dentin matrix/α-calcium sulphate hemihydrate composite material for bone tissue engineering
Source: Regen Biomater. 2023 Jun 19;10:rbad061. doi: 10.1093/rb/rbad061 (PMC10369214; doi:10.1093/rb/rbad061)
Supplement: rbad061_Supplementary_Data [file rbad061_supplementary_data.docx]

**Biomimetic, biodegradable, and osteoinductive treated dentin matrix/α-calcium sulphate hemihydrate composite material for bone tissue engineering**

Runying Guo ^1,2,†^, Rui Zhang ^3,†^,Sirui Liu ^1^ , Yanyu Yang ^4^ , Wenhang Dong ^1^ , Meiyue Wang ^1^ , Hongyan Mi ^1^ , Mengzhe Liu ^1^ , Jingjing Sun ^1^ , Xue Zhang ^1^ , Yimeng Su ^5^ , Yiming Liu ^1,*^, Di Huang ^5,*^, Rui Li ^1,*^

^1^Department of Stomatology, The First Affiliated Hospital of Zhengzhou University,

Zhengzhou 450052, PR China

^2^Department of Stomatology, The First Affiliated Hospital of Nanchang University,

Nanchang 330052, PR China

^3^Oral and maxillofacial departement, Zhengzhou Stomatology Hospital, Zhengzhou

450099, PR China

^4^College of Materials Science and Engineering, Zhengzhou University, Zhengzhou

450001, PR China

^5^Research Center for Nano-biomaterials and Regenerative Medicine, College of

Biomedical Engineering, Taiyuan University of Technology, Taiyuan 030024, PR

China.

† These authors contributed equally to this work

^*^Correspondence addresses. Email: fcclir@zzu.edu.cn (R.L.); doctorliuym@163.com (Y.L.); huangdi@tyut.edu.cn (D.H.)


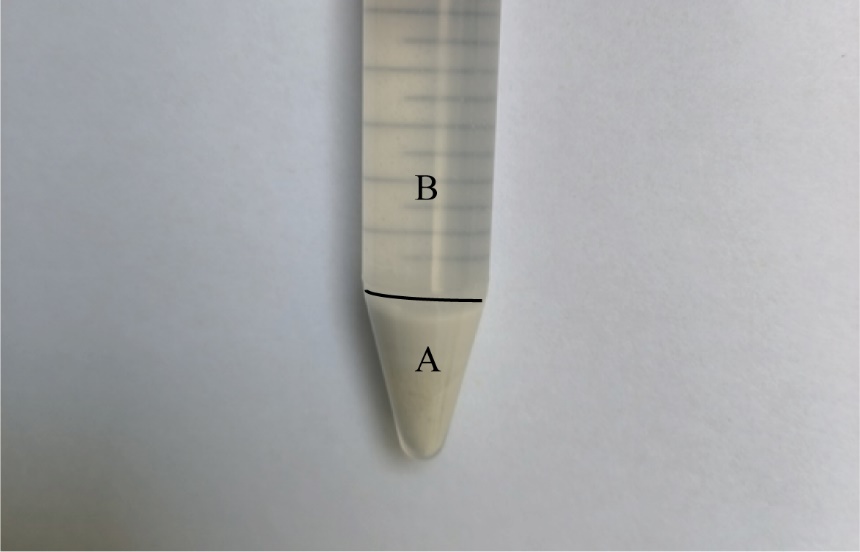


Supplementary Figure S1. Visual observation of CSH after contact with simulated body fluids. (A：70%TDM/α- CSH, B: Simulated body fluids)


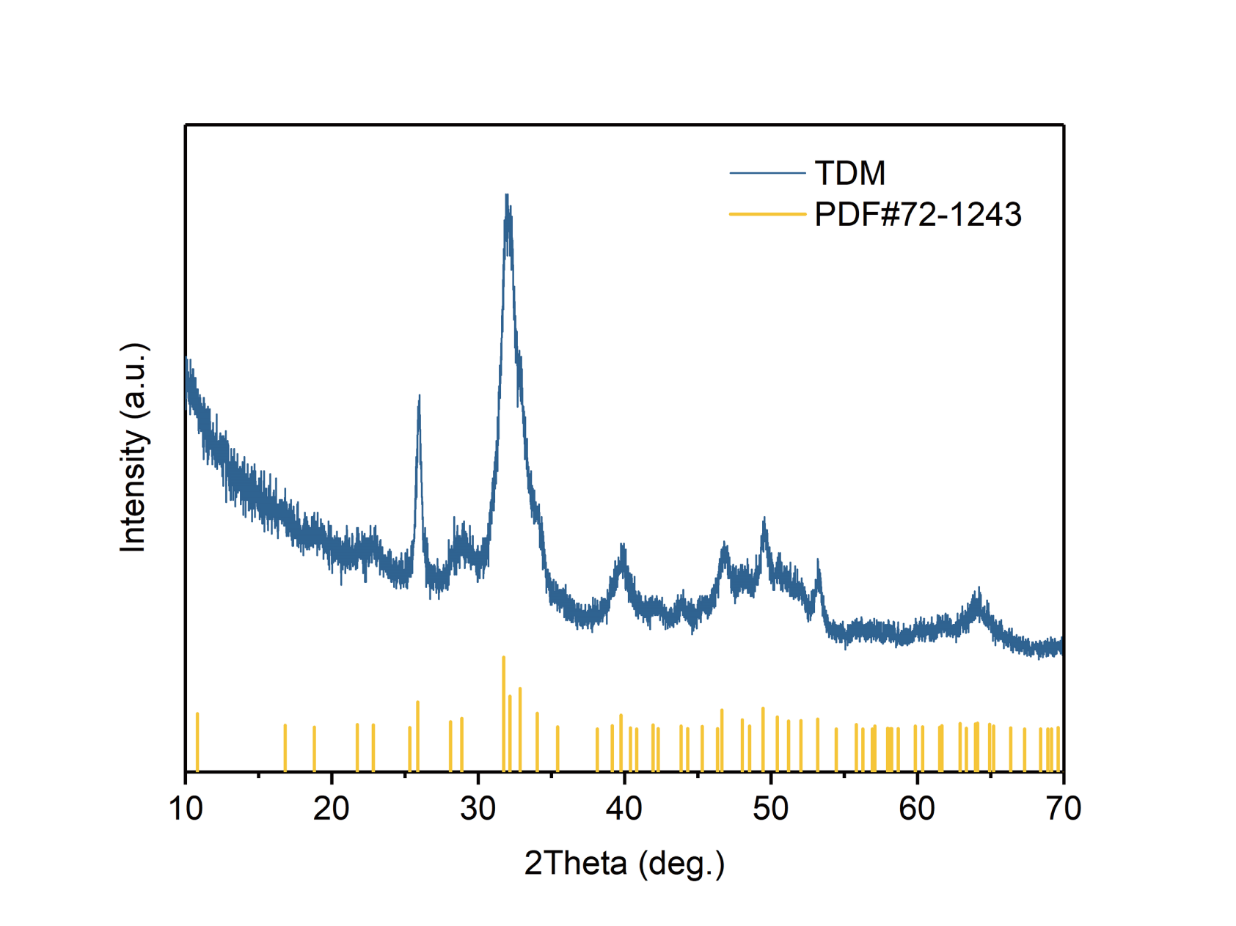


Supplementary Figure S2.The TDM material was evaluated based on the occurrence of standard HA diffraction peaks (PDF: 42-1243); thus, peaks at 35°, 39°–41°, 45.5°–53°, and 54°–65° were found to be attributable to the TDM material. The broad and strong diffraction peaks prove that the crystallinity of the TDM was lower than that of the synthetic HA.


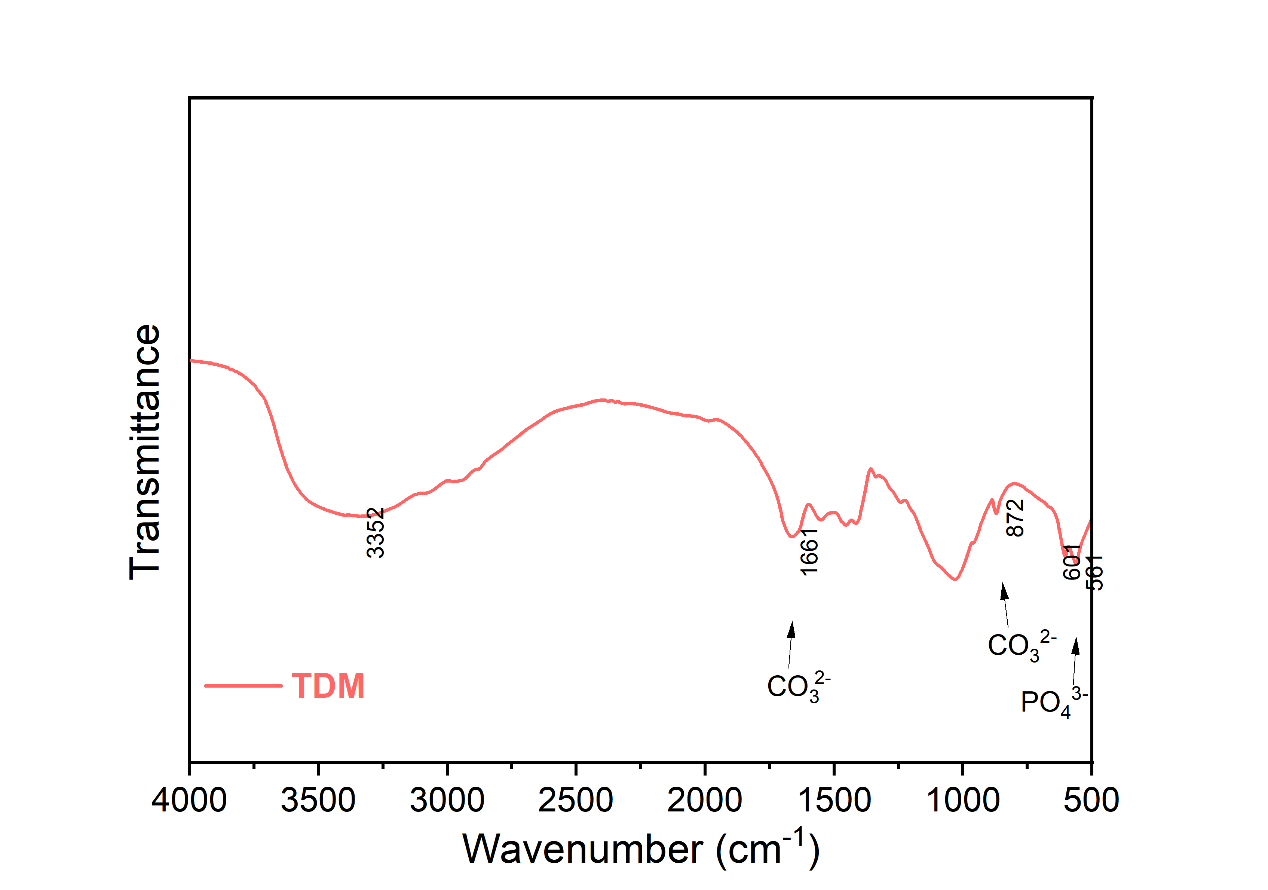


Supplementary Figure S3. The FTIR detection results were consistent with those of XRD analysis, further verifying that the main component of the TDM was HA that contained organic groups such as CO_3_^2-^ and PO_4_^3-^ groups.


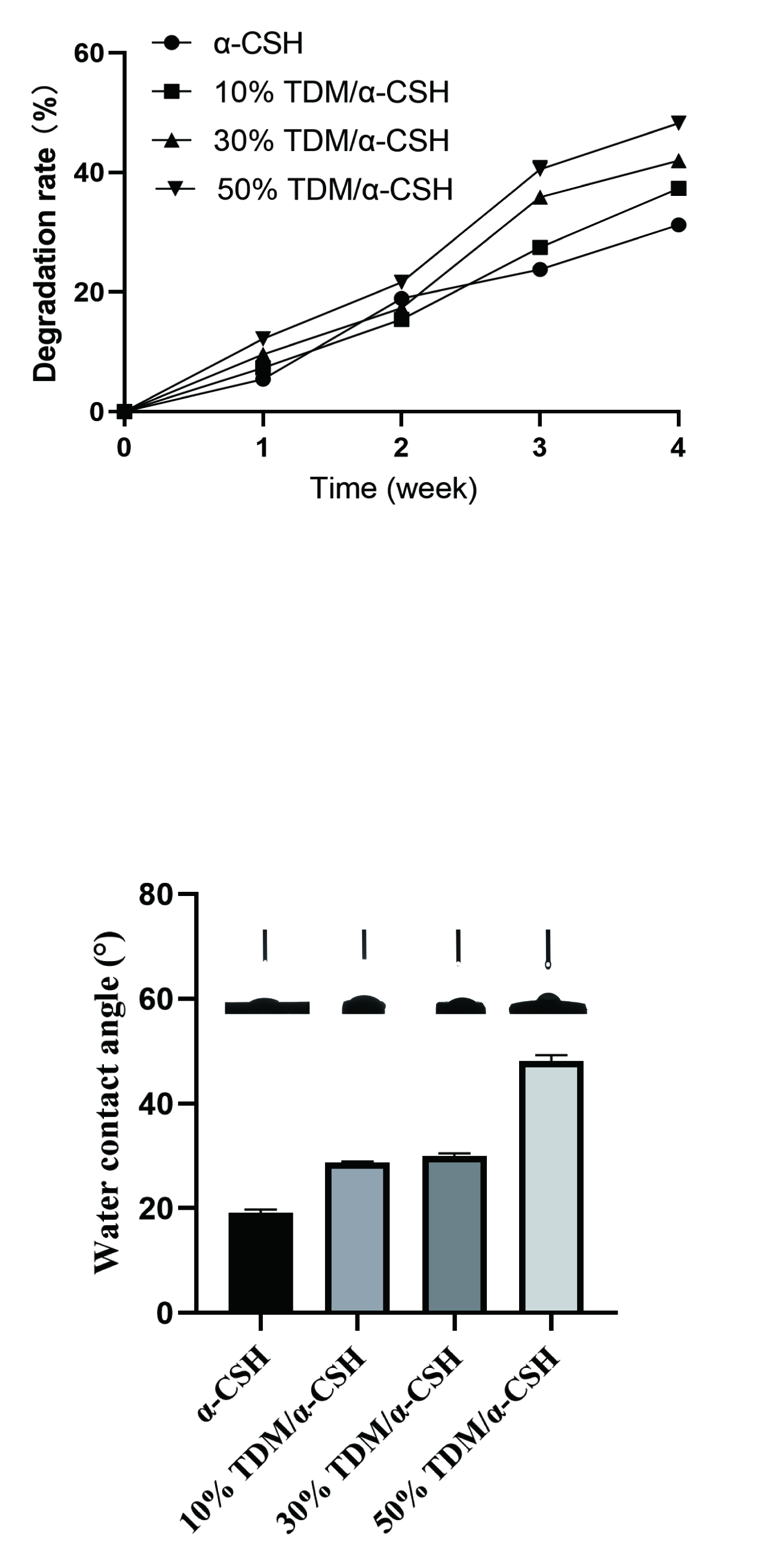


Supplementary Figure S4. The degradation experiment demonstrates that as the content of TDM increasing, the rate of degradation of the composite material increases.


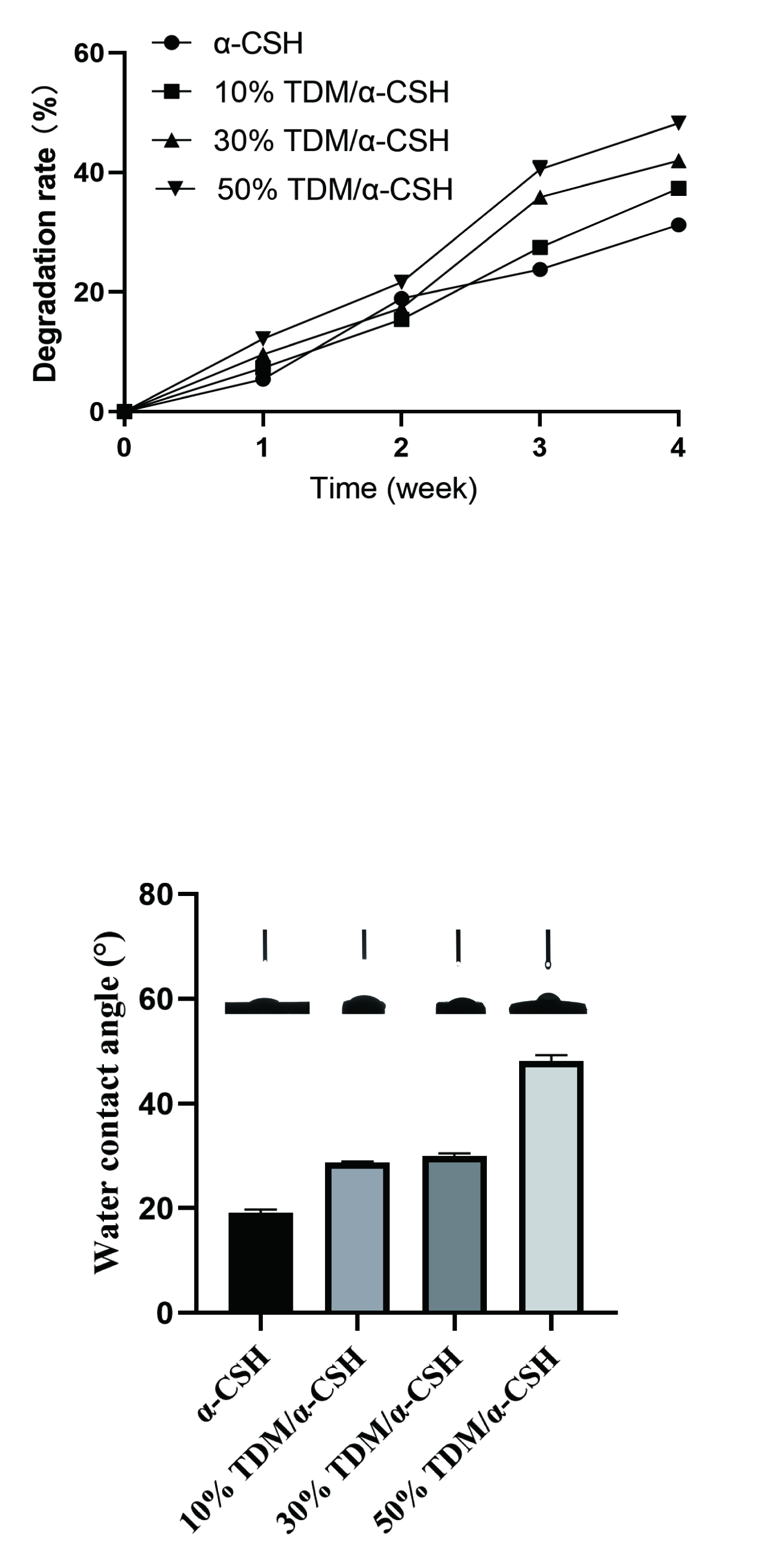


Supplementary Figure S5. The contact angles of each group of materials indicate that all samples present hydrophilic property.
